# Supplementary material for: The causal role of circulating inflammatory markers in osteoporosis: a bidirectional Mendelian randomized study
Source: Front Immunol. 2024 Jul 18;15:1412298. doi: 10.3389/fimmu.2024.1412298 (PMC11291241; doi:10.3389/fimmu.2024.1412298)
Supplement: Supplementary file 1 [file DataSheet_1.zip › supplementary material/OSTEOPOROSIS-CIM/GCST90274837.tsv.gz_leave_one_out.pdf]

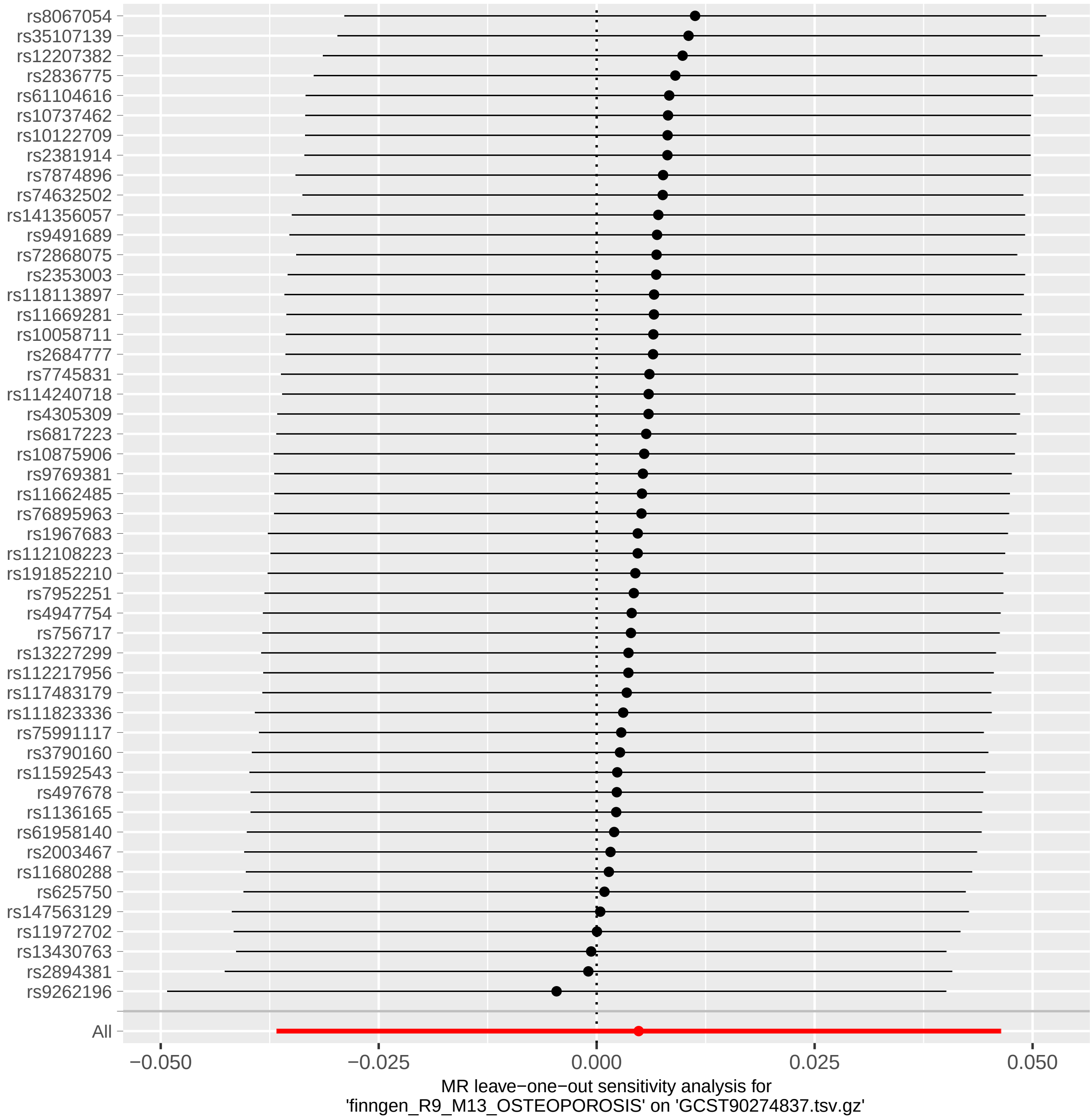

rs8067054
rs35107139
rs12207382
rs2836775
rs61104616
rs10737462
rs10122709
rs2381914
rs7874896
rs74632502
rs141356057
rs9491689
rs72868075
rs2353003
rs118113897
rs11669281
rs10058711
rs2684777
rs7745831
rs114240718
rs4305309
rs6817223
rs10875906
rs9769381
rs11662485
rs76895963
rs1967683
rs112108223
rs191852210
rs7952251
rs4947754
rs756717
rs13227299
rs112217956
rs117483179
rs111823336
rs75991117
rs3790160
rs11592543
rs497678
rs1136165
rs61958140
rs2003467
rs11680288
rs625750
rs147563129
rs11972702
rs13430763
rs2894381
rs9262196
All
−0.050
−0.025
0.000
0.025
0.050
MR leave−one−out sensitivity analysis for
'finngen_R9_M13_OSTEOPOROSIS' on 'GCST90274837.tsv.gz'
